# Arthroscopically assisted (AORIF) ankle fracture treatment seems to lead to superior results when compared to open reduction and internal fixation (ORIF) only: results of a systematic review

**DOI:** 10.1007/s00402-025-06030-4

**Published:** 2025-09-18

**Authors:** Rainer Christoph Miksch, Fabian Tobias Spindler, Wolfgang Böcker, Hans Polzer, Sebastian Felix Baumbach

**Affiliations:** LMU Klinikum, Munich, Germany

**Keywords:** Arthroscopy, Ankle fracture, PROM

## Abstract

**Introduction:**

Ankle fractures often involve intra-articular pathologies, which can only be addressed by additional arthroscopy. This systematic review aims to compare the outcomes of arthroscopically assisted open reduction and internal fixation (AORIF) with traditional open reduction and internal fixation (ORIF) for ankle fractures.

**Materials and methods:**

A systematic literature search adhering to PICOS and PRISMA guidelines was conducted across the following databases: MEDLINE (PubMed), Scopus, Central and EMBASE. Studies that compared AORIF and ORIF of ankle fractures and focused on patient-reported outcome measures (PROMs) as the primary outcome were included. Excluded were studies on non-acute or non-isolated fractures, pilon fractures, concomitant injuries outside the ankle, biomechanical or computational studies, and those lacking objective outcome data.

**Results:**

A total of 7089 studies were screened, 12 of which met the inclusion criteria for qualitative synthesis. The level of evidence was I-III with a mean MINORS Tool score of 19.17. Among the included studies, six studies focused on unimalleolar fractures, with four demonstrating significantly better PROMs for the AORIF group. Five studies addressed bimalleolar and/or trimalleolar fractures, with one showing significantly better PROMs for AORIF. Seven studies reported on intra-articular pathologies, with a detection rate of up to 88.89%. Two out of three studies on posttraumatic arthritis indicated lower grades of osteoarthritis in the AORIF group.

**Conclusion:**

The review suggests that AORIF may lead to improved scores as obtained through various PROMs compared to ORIF, particularly for unimalleolar fractures. However, the heterogeneity among the underlying studies indicates the need for further research to identify specific patient populations and fracture types that would benefit the most from AORIF.

**Supplementary Information:**

The online version contains supplementary material available at 10.1007/s00402-025-06030-4.

## Introduction

Ankle fractures are among the most common fractures in adults, accounting for a significant proportion of orthopedic injuries [1, 2]. Ankle fractures have been reported to result in impaired patient-rated outcomes, leading to a reduced quality of life for patients [2, 3]. Risk factors for an impaired patient-reported outcome can be broadly classified into non-modifiable and potentially modifiable factors.

Non-modifiable risk factors include fracture severity [3, 4, 5], BMI [6], age and gender [2, 7]. Modifiable risk factors include the quality of reduction [8] as well as the identification and treatment of accompanying ligamentous and intra-articular injuries [9]. The latter represent the only possibility of improving the patient-rated outcome.

The current paper focuses on the identification and treatment of accompanying intra-articular pathologies. Their identification remains a considerable challenge. Studies have indicated that even magnetic resonance imaging (MRI) has limited efficacy in identifying intra-articular lesions in acute ankle fractures [10, 11, 12]. The gold standard in the diagnosis of intra-articular pathologies is arthroscopy [13, 14]. Arthroscopy not only enables the diagnosis of intra-articular pathologies but also their treatment in the same procedure. Consequently, arthroscopically assisted open reduction and internal fixation (AORIF) could be a promising approach to improve the patient-rated outcome in ankle fracture cases.

Still, American registry studies have shown that less than 1% of ankle fracture treatments include arthroscopy [15, 16, 17]. Possible explanations for the missing acceptance of AORIF include a fear of increased complication rates, longer operative durations and higher costs.

We hypothesize that arthroscopically assisted open reduction and internal fixation (AORIF) leads to comparable patient-reported outcomes compared to traditional open reduction and internal fixation (ORIF) in patients with ankle fractures, without increasing complication rates or significantly prolonging operative time.

Consequently, the aim of this systematic review was to evaluate and compare the clinical outcomes of AORIF versus ORIF in the treatment of ankle fractures.

## Materials and methods

This systematic review was conducted according to the Preferred Reporting Items for Systematic Reviews and Meta-Analyses (PRISMA) guidelines [18]. It was registered, a priori, to PROSPERO [CRD42022308275] in February 2022 and updated in January 2025.

### Search strategy

**Fig. 1:**
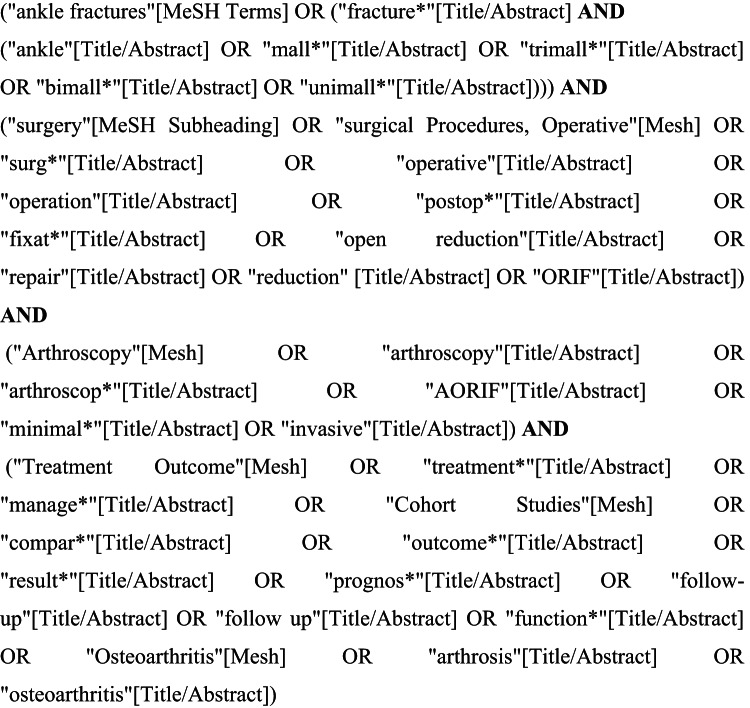
Search strategy for MEDLINE (PubMed)

The databases that were searched included MEDLINE (PubMed), Scopus, Central and EMBASE, with search parameters stretching from inception to January 2025. The search strategy was built for MEDLINE (Fig. 1) and adapted accordingly. It comprised of the concepts “ankle fracture” AND “surgery” AND “arthroscopy” AND “outcome”. The individual search strategies are presented in Suppl 1. A grey literature search for conference proceedings in both Scopus and EMBASE was performed.

## Eligibility criteria

The inclusion criteria were framed according to the Patient, Intervention, Comparison, Outcome and Study design (PICOS) and framework. Eligible were studies involving (P) skeletally mature patients with an acute, isolated ankle fracture that compared (I) arthroscopically assisted open / closed reduction and internal fixation (AORIF) to (C) open / closed reduction and internal fixation (ORIF). Acute was defined as patients receiving fracture treatment within the first 3 weeks of injury. Isolated was defined as an isolated ankle fracture without concomitant injuries outside the ankle joint. Ankle fractures were classified as uni-, bi-, or trimalleolar fractures, including Wagstaff or Tubercule de Chaput fractures, irrespective of the associated ligamentous injuries, i.e. injuries to the syndesmotic or deltoid ligament complex. Patients were allowed to be initially stabilized by an external fixation. Studies had to report on at least one (O) objective outcome parameter, such as a patient-reported outcome measurement (PROM), quality of reduction, function or osteoarthritis. Studies needed to present the outcome data separately for the different treatment groups. Eligible were (S) any original, comparative studies written in English, German, French, Spanish or Portuguese.

Exclusion criteria were ankle fractures that were neither acute nor isolated, concomitant fractures, pilon fractures, missing objective outcome parameters or biomechanical / computational studies.

The year of publication was not an exclusion criterion.

## Study selection and data extraction

The entire study selection and data extraction process was conducted by two independent reviewers (RCM, FTS). The final data extraction was performed in February 2025. In case of dissent, a third reviewer (SFB) resolved the conflict in discussion. The individual search results from each database were imported into Endnote (X9.3.3, Clarivate [19]) and duplicates were removed using the Endnote standard algorithm. The final dataset was imported to Covidence systematic review software (Veritas Health Innovation, Melbourne, Australia). The further study selection process was performed within Covidence. First, the titles and / or abstracts were screened. In case of doubt, the study was handed to the full-text review section. Second, a full-text review was performed.

Data extraction and quality rating of the eligible studies were conducted on separate excel sheets. Fractures were classified per the number of bony fractures into unimalleolar, bimalleolar and trimalleolar ankle fractures. The Lauge-Hansen classification was converted into the above-outlined classification. Ligamentous injuries were not regarded in the classification process.

## Risk of bias assessment

The level of evidence was assessed independently by two of the authors according to the rating system introduced by Wright et al. (RCM, SFB) [20]. Disagreements were resolved by discussion. The methodological quality was assessed using the Methodological Index for Non-Randomized Studies (MINORS) [21]: The items were scored 0 (not reported), 1 (reported but inadequate) or 2 (reported and adequate). The ideal score is 16 points for non-comparative studies and 24 points for comparative studies and has been validated for randomized studies and nonrandomized studies (Suppl Table 1).

## Data synthesis and analysis

The primary outcome parameter was any patient-reported outcome measure (PROM). Secondary outcome parameters were any other objective outcome measures. Data synthesis is presented descriptively. If three or more studies are of sufficient equality/comparability, a meta-analysis can be conducted using ReviewManager (Version 5.4, The Cochrane Collaboration).

## Results

**Fig. 2:**
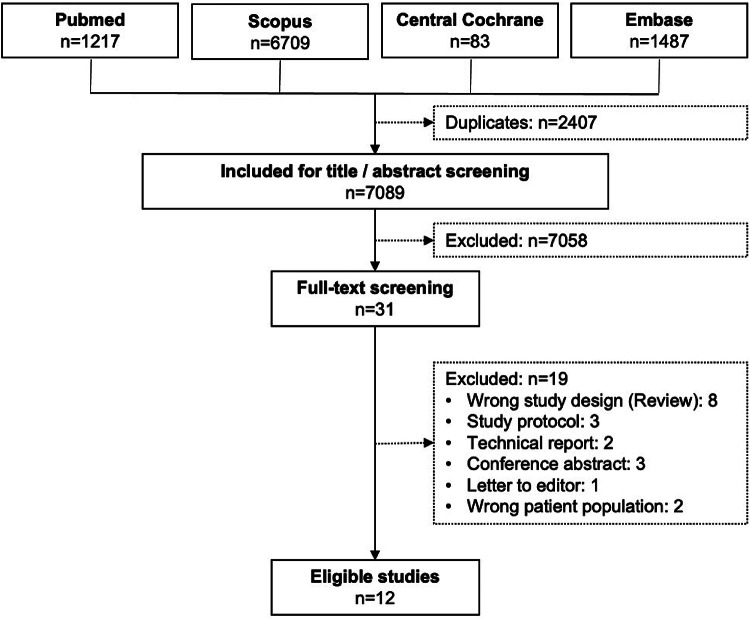
PRIMSA flow chart. n = number of studies

The PRISMA flow chart is presented in Fig. 2. In summary, out of 7089 studies screened for title / abstract, 31 studies were included in the full-text review and twelve studies were eligible for the qualitative synthesis [16, 22, 23, 24, 25, 26, 27, 28, 29, 30, 31, 32]. The studies characteristics are outlined in Table 1.

## Overall study characteristics

The overall study characteristics are outlined in Table 1. Three studies were randomized controlled trials (RCTs), two were prospective and seven were retrospective cohort studies. The mean study quality as per the MINORS criteria was 19.17 (Supp. Table 1) [20, 21]. On average the studies comprised 73.83 ± SD 52.26 (19–213) patients, with a mean age ranging from 34 to 65.8 years (AORIF) and 38 to 66.5 years (ORIF) and a follow-up duration ranging between 6.4 and 67 months. In total, twelve different **PROMs** were used in the 12 studies included. Eleven studies reported on peri-/postoperative **complications** [16, 22, 23, 24, 25, 26, 27, 28, 29, 30, 32], with ten studies differentiating between the treatment applied (AORIF vs. ORIF) [16, 22, 23, 24, 26, 27, 28, 29, 30, 32]. The overall complication rates did not differ between AORIF (average 6% (0%-13.6%)) and ORIF (average 9% (0%-28%)). Eight studies reported on the **duration of the operation** (Table 5) [23, 24, 25, 26, 27, 30, 31, 32]. In 7/8 studies AORIF took significantly longer, on average 17.29 min.

The overall heterogeneity between the studies, as per the authors’ perception, did not allow for a meta-analysis. For further qualitative analysis, the studies were grouped according to the severity of the ankle fractures included, i.e. unimalleolar vs. bi-/trimalleolar ankle fractures. This was done as fracture severity has been shown to be one of the most influential parameters on the patient-rated outcome [1].

## Quality of the included studies

The level of evidence defined by Wright et al. 2003 was between I, II and III in the included studies (Suppl. Table 1). Three studies were level of evidence I and defined as randomized controlled trials in Table 1, one study was level II and the other eight studies were level III studies. Actually, two studies were prospective studies (Table 1). The rest of the included studies were retrospective studies. Following the MINORS Tool, the mean score was 19.17. One study by Liu et al. reached the maximum score of 24. All other studies revealed a global ideal score > 16.

### Unimalleolar ankle fractures

Six studies reported solely on unimalleolar ankle fractures [26, 27, 28, 29, 30, 31]. Three studies were RCTs [28, 29, 31], one study was a prospective [27] and two studies were retrospective cohort studies [26, 30]. On average the studies comprised 55.67 ± SD 21.52 (range 19–77) patients with a mean age ranging from 34 to 65.8 years (AORIF) and 38 to 66.5 years (ORIF) and a follow-up duration ranging between 21 and 67 months.

Arthroscopy was performed through standard anteromedial and -lateral portals in five studies [26, 27, 28, 29, 30]. Only Ge et al. [31] undertook a posterolateral approach for the ankle arthroscopy. The primary intention of the arthroscopy was the detection of intra-articular pathologies in three studies [26, 28, 29], while the remaining three studies aimed to assess the quality of reduction [27, 30, 31].

Overall, 4/6 studies reported significantly better **PROMs** for the AORIF cohort [27, 28, 30, 31] (Table 2). The remaining two studies found no significant differences between the AORIF and ORIF cohorts [26, 29].

5/6 studies reported on the **complication rates** [26, 27, 28, 29, 30] (Table 4), which did not differ significantly between the two groups and were, on average, 3% for AORIF and 5% for ORIF. The remaining study did not statethe complication rate [31].

4/6 studies reported on the **duration of surgery** [26, 27, 30, 31] (Table 5), which was significantly longer for the AORIF group, on average 16 min, in three studies [REF].

### Bi- and trimalleolar ankle fractures

The other six studies reported on bi-/trimalleolar (Table 3) ankle fractures (also classified according to SER and Lauge-Hansen ) [16, 22, 23, 24, 25, 32]. All six studies were retrospective [16, 22, 23, 24, 25, 32], with five studies facilitating a retrospective cohort study design [22, 23, 24, 25, 32] and one study comparing a prospective AORIF to a retrospective ORIF cohort [16]. On average the studies comprised 92 ± SD 68.94 (range 22–213) patients with a mean age ranging from 32.82 to 50.9 years (AORIF) and 32.82 to 53.3 years (ORIF) and a follow-up duration ranging between 6.4 and 52.8 months.

In 5/6 studies the intention for arthroscopy was the identification of intra-articular lesions [16, 22, 24, 25, 32], while the remaining study aimed to assess the quality of reduction [23].

Overall, 2/6 studies [16, 32] showed significantly improved **PROMs** for the AORIF groups. On the contrary, no study showed significantly improved parameters for the ORIF group.

All 6 studies reported on the **complication rates** (Table 4), which did not differ significantly between the two groups and were, on average, 9% for AORIF and 14% for ORIF. Smith et al. reported a combined (AORIF + ORIF) complication rate of 8% [25].

4/6 studies reported on the **duration of surgery** [23, 24, 25, 32] (Table 5), which was significantly longer in the AORIF group in two studies [23, 24]. Chou et al. [32] was the only study that reported a significantly longer duration of surgery for the ORIF group, which was not explained.

## Discussion

the aim of this systematic review was to evaluate and compare the clinical outcomes of AORIF versus ORIF in the treatment of ankle fractures. While previous systematic reviews have suggested potential benefits of AORIF, our study adds depth by stratifying findings according to fracture severity and by critically analyzing the context and limitations of available evidence. A key observation from our analysis is that the anticipated advantage of AORIF in more complex fractures (bi-/trimalleolar) was not clearly supported by the data. In fact, significantly better patient-reported outcomes (PROMs) were more frequently observed in unimalleolar fractures, with 4 out of 6 studies demonstrating superior outcomes for AORIF in this subgroup, compared to only one study in the bi-/trimalleolar group. This finding is particularly noteworthy as previous literature has associated more severe fractures with greater intra-articular damage, suggesting they should benefit more from arthroscopic intervention. The inverse trend observed may be explained by the higher overall trauma burden and increased confounders in complex fractures, which could mask the potential benefits of AORIF. Conversely, simpler fracture patterns may allow the effect of arthroscopy to be more clearly observed due to fewer competing variables. The quality of evidence also differed between groups. Studies on unimalleolar fractures were generally of higher methodological quality, with a greater proportion of prospective designs. This may partially account for the more consistent positive outcomes in this subgroup, but it also raises important questions about the generalizability of findings in more severe fracture patterns, where fewer high-quality studies are available.

Intra-articular pathologies frequently occur in ankle fractures [28, 29], they are best diagnosed by arthroscopy [13, 14] and are believed to have a negative impact on the outcome [11, 33]. The intraoperative and diagnostic accuracy of arthroscopy outweighs the current capabilities of MRI due to the slice thicknesses involved in diagnosing intra-articular lesions. This is especially true since intraoperative diagnostics can also directly lead to therapeutic options.

Consequently, the authors assumed that the predominant reason for AORIF would be the identification and treatment of these accompanying intra-articular pathologies. Surprisingly, three studies specifically stated that the primary motivation for additional arthroscopy was to assess the quality of reduction [27, 30, 31]. Still, in the authors’ practice the primary rationale for AORIF is the diagnosis and treatment of intra-articular pathologies. Secondary rationales are the assessment of syndesmotic and deltoid ligament injuries as well as the quality of reduction, especially of the posterior and medial malleoli. Following the literature, AORIF is primarily indicated for the detection and management of intra-articular pathologies, such as osteochondral lesions and loose bodies, which are often missed by conventional imaging but can negatively impact patient outcomes. Additionally, arthroscopy provides direct visualization of the joint surface, allowing for more precise assessment and improvement of fracture reduction, particularly in complex fracture patterns involving the posterior or medial malleoli. It is also valuable for evaluating and treating associated ligamentous injuries, including syndesmotic and deltoid ligament damage, which contribute to joint stability. While AORIF is commonly considered in complex bi- or trimalleolar fractures, recent evidence suggests it may offer particular benefits even in unimalleolar fractures by addressing occult intra-articular injuries that could otherwise impair recovery which was also shown for isolated syndesmotic injuries.

In regard to the primary outcome of this systematic review, the patient-rated outcome, 5/12 studies reported significantly better PROMs for AORIF compared to ORIF. No study found superior outcomes for the ORIF group. Similar findings were reported by three recent systematic reviews [13, 14, 34]. All of these studies performed meta-analyses and reported significantly superior functional outcomes, as measured by OMAS and VAS scores, in the AORIF groups. Based on these findings, they suggested a potential clinical benefit of AORIF. In contrast, the present review deemed the available evidence too heterogeneous to permit a meta-analysis. Furthermore, prior systematic reviews have not adequately identified specific patient subgroups that may derive the greatest benefit from the addition of arthroscopy.

The current systematic review is the first to separately analyze the available evidence according to fracture severity, i.e. uni- vs. bi-/trimalleolar ankle fractures. As previous studies have shown a correlation between the severity of intra-articular pathologies and fracture severity [16, 33, 35], the authors initially expected a more prominent effect of AORIF on more complex injuries (bi-/trimalleolar). Interestingly, this systematic review yielded contrasting findings: significantly better outcomes for AORIF compared to ORIF were reported in 4 out of 6 studies focusing on unimalleolar fractures, whereas only 1 out of 6 studies addressing bi- and trimalleolar fractures demonstrated such benefits. Additionally, the level of evidence was generally higher among studies investigating unimalleolar fractures, with four of six being prospective in design, compared to only one of six prospective studies in the bi-/trimalleolar fracture group. A plausible explanation for these findings is that the greater overall trauma associated with bi- and trimalleolar fractures may overshadow the potential benefits of adjunctive arthroscopy, rendering its effect less pronounced amid other confounding factors.

Whenever assessing PROMs one must keep in mind that statistical differences do not necessarily represent clinically relevant findings, i.e. exceed the minimal clinically important difference (MCID). 2/4 studies reporting on the OMAS [16, 26, 27, 30] found statistically significant better results for the AORIF cohort [16, 30]. However, the difference exceeded the MCID of 10 points [36, 37] in only one of the studies [16]. The same is true for the studies using the AOFAS [23, 32, 38]. These findings highlight the importance of critically analyzing the evidence available [39].

Common criticisms of adjunctive arthroscopy include concerns about increased complication rates and prolonged operative times. Both of these factors were evaluated in the present systematic review. Importantly, none of the included studies demonstrated a significant increase in overall mean complication rates for AORIF (average 6%, range 0–13.6%) compared to ORIF (average 9%, range 0–28%). These results align with findings from a large database analysis of 32,307 ankle fractures, which similarly reported no increase in reoperation or complication rates associated with the addition of arthroscopic procedures [40]. Nevertheless, the herein-conducted analysis was limited due to the fact that sufficient detail on the actual complications was missing, to differentiate, for example, between minor and major complications. Moreover, patients must be informed that additional ankle arthroscopy has specific complications, notably a (temporary) lesion to the N. peroneus comm. sup. In a current systematic review severe, life-threatening complications are rare, occurring in just 0.2% of patients, with most complications being minor. Although AORIF does not seem to increase the overall risk of complications, surgeons must weigh the inherent complications of additional arthroscopy against the benefit of identifying and treating intra-articular pathologies.

The second point of criticism, i.e. an increase in operation time, was proven right. Additional arthroscopy increased the overall duration of surgery by 17 min on average. Although not explicitly stated, these 17 min most likely do not include the additional pre-incision preparation time. Whereas this preparation time cannot be significantly influenced, the increase in operation time varies depending on the intra-articular pathologies found. With that said, on the one hand one can assume that there is a learning curve, on the other hand the operative time will increase if additional arthroscopic procedures, such as microfracturing, have to be performed. These should also hopefully result in a superior long-term outcome [40]. Furthermore, additional arthroscopy does not lead to significant cost in an US-based study population.

The heterogeneity of the included studies remains the primary limitation of this review. Variations in study design, surgical technique, outcome measures, and follow-up duration hinder direct comparisons and preclude meta-analysis. The include studies were published between 2001 and 2023. As bi- and trimalleolar ankle fractures were also added in this systematic review, it should be considered the treatment options in general but especially of the posterior malleolar fracture are still evolving. Additionally, the average follow-up, in the studies included, ranged between 6.4 and 61.5 months. Consequently, we are still lacking long-term follow-up data to assess the impact of AORIF on the development of posttraumatic arthritis. Long-term follow-up plays a critical role in the management and evaluation of ankle fractures, as it provides essential insights into the progression of posttraumatic complications, particularly the development of osteoarthritis and chronic functional impairments. While short- to mid-term outcomes often focus on fracture healing and initial functional recovery, it is the long-term data that ultimately reflect the durability of treatment effects and the patient’s quality of life over time. Another critical limitation is the underreporting of postoperative reduction quality—only two studies explicitly assessed this—which may have influenced outcomes independent of the surgical approach used [16, 23]. To better evaluate the effect of additional procedures, i.e. arthroscopy, one must strive to reduce secondary confounders, such as a non-anatomical fracture reduction.

This review distinguishes itself from prior analyses through its rigorous adherence to PRISMA guidelines and registration with PROSPERO, thereby ensuring methodological transparency and minimizing bias. It offers a clear and consistent classification of fracture types, specifically differentiating between unimalleolar and bi-/trimalleolar fractures. Furthermore, the detailed description of data collection and outcome measures facilitates a robust synthesis of findings by including only high-quality studies featuring well-defined endpoints and appropriately matched comparative groups.

## Conclusion

Arthroscopically assisted ORIF (AORIF) appears to offer superior patient-reported outcomes in unimalleolar ankle fractures, particularly in studies with higher methodological quality. However, its added value in more complex bi-/trimalleolar fractures remains uncertain. Importantly, AORIF was not associated with increased complication rates but did lead to a modest increase in operative time. These findings suggest that the benefit of AORIF may be fracture-type dependent and potentially influenced by case complexity and surgical expertise. Future high-quality, prospective studies with standardized outcome reporting and long-term follow-up are essential to better define the role of AORIF in ankle fracture management and to identify patient subgroups most likely to benefit.

**Table 1:** Overview of the twelve studies included in this systematic review, comprising randomized controlled trials (RCTs), prospective, and retrospective studies

Authors	Year	Fracture type	Treatment	Number of patients	Follow-up [months]	FU *p* value	MINORS score
RCT							
Ge et al.	2017	Unimall	AORIF red.* ORIF	34 34	NA		18
Takao et al.	2004	Unimall	AORIF ORIF	41 31	40 41		18
Thordarson et al.	2001	Unimall	AORIF ORIF	9 10	21		17
Prospective study							
Baumbach et al.**	2021	Bi-/Trimall	AORIF ORIF	25 25	52.8 48.0	*p* = 0.064	20
Liu et al.	2020	Unimall	AORIF red. ORIF	34 43	60.1 61.5	*p* = 0.634	24
Retrospective studies							
Ceccarini et al.	2021	Bi-/Trimall	AORIF ORIF	38 75	38.1 40.1	NA	20
Chiang et al.	2019	Bi-/Trimall	AORIF red. ORIF	65 40	40.0 38.4	*p* = 0.597	20
Chou et al.	2023	Bi-/Trimall	AORIF ORIF	22 27	36.4	*p* = 0.114	20
Danilkowicz et al.	2021	Bi-/Trimall	AORIF ORIF	11 11	8.2 6.4	NA	19
Fuchs et al.	2016	Unimall	AORIF ORIF	24 27	67		20
Smith et al.	2020	Bi-/Trimall	AORIF ORIF	71 142	32.4		18
Turhan et al.	2012	Unimall	AORIF red. ORIF	21 26	26 38		16

Follow-up (FU) durations are presented in months. NA = not available. *Studies highlighted with “red.” indicate those that employed additional arthroscopy primarily to verify fracture reduction. **Matched-pair analysis

**Table 2:** Unimalleolar fractures: the primary purpose of arthroscopy in these studies was to detect intra-articular pathologies

Authors	Detection of cartilagenous pathologies	PROMs	PROMs AORIF vs. ORIF	*P* value	Posttraumatic arthritis
Fuchs et al.	*	PROMIS pf	57.8 vs. 54.5	0.23	NA
		PROMIS pain	45.6 vs. 46.9	0.56	
		OMAS	90 vs. 84	0.11	
		VAS	1.0 vs. 2.1	0.13	
Ge et al.	NA	McGuire	86.5 vs. 71.5	**0**	NA
		VAS	5.5 vs. 4.1	**0**	
		Therapeutic effects	91.2% vs. 70.6%	**0**	
Liu et al.	32.40%	ROM DE	20.6 vs. 19.9	0.354	0
		ROM PF	46.2 vs. 43.8	**0.001**	7% (3x grade I van Dijk classification)
		OMAS	97.9 vs. 96.6	0.081	
Takao et al.	73.20%	AOFAS	91.0 vs. 87.6	**0.011**	NA
Thordarson et al.	88.89%	SF-36	Multiple scoring	NA	NA
		MODEMS	Multiple scoring		
Turhan et al.	7x grade I/II ICRS	OMAS	92.3 vs. 86.3	**0.015**	1x grade I van Dijk
					3x grade I/II

Studies where arthroscopy was additionally used to assess the quality of reduction are marked with “red.” under “AORIF”. However, no data regarding reduction quality control were available for these studies. Notably, 62% (26 of 42) of patients treated with arthroscopy had articular surface lesions. PROMIS Physical Function (PF) scores were reported. Turhan et al. included only isolated medial malleolar fractures. Patient-reported outcome measures (PROMs) comparing AORIF and ORIF were analyzed, with corresponding p-values provided

**Table 3:** Bi- and trimalleolar fractures: significant p-values are highlighted

Authors	Detection of cartilagenous pathologies	Control of reduction	*P* value	PROMs	PROMs AORIF vs. ORIF	*P* value	Posttraumatic arthritis
Baumbach et al.	NA	100%	0.369	OMAS	90 vs. 75	**0.008**	NA
				FAAM	96 vs. 88	**0.034**	
				FAAM sports score	88 vs. 56	**0.008**	
Ceccarini et al.	NA	NA		ROM	*		No sig.
				FAOS	Sub-scores described		
Chiang et al.	NA	NA		VAS	0.75 vs. 1.12	0.141	NA
				AOFAS	92.34 vs. 89.79	0.084	
				ROM PF	46.05 vs. 44.16	0.447	
				ROM DE	16.30 vs. 16.00	0.837	
				ROM total	62.34 vs. 60.16	0.482	
Chou et al.	NA‘	100%	0.375	VAS	0.8 vs. 1.1	0.191	0.368
				AOFAS	90.8 vs. 85.8	0.139	
				ROM PF	54 vs. 50	0.279	
				ROM DE	13 vs. 11	0.122	
				ROM Total arc	66 vs. 61	0.132	
				ROM Inversion	18 vs. 15	**0.033**	
				ROM Eversion	7 vs. 8	0.26	
Danilkowicz et al.	NA	NA		VAS	0.7273 vs. 0.8182	0.8939	NA
Smith et al.	77%	NA		PROMIS physical function	44.9 vs. 72.7	0.064	NA

Range of motion (ROM) measures include plantarflexion (PF) and dorsiflexion (DF); data marked as not available (NA) where not applicable. Cartilaginous pathologies were identified and treated, although exact numbers were not reported. *Differences in changes of PF and DF were described, including comparison with a third study group

**Table 4:** Complication rates are reported for each study, including comparisons between arthroscopically assisted open reduction and internal fixation (AORIF) and open reduction and internal fixation (ORIF)

Author	Study design	Patients	Number	Complications	*P* value
Unimalleolar fractures					
Ge et al.	RCT	AORIF red ORIF	34 34	NA NA	
Takao et al.	RCT	AORIF ORIF	41 31	0% 7%	NA
Thordarson et al.	RCT	AORIF ORIF	9 10	0% 0%	NA
Liu et al.	Prospective	AORIF red ORIF	34 43	8.8% 18.6%	0.329
Fuchs et al.	Retrospective	AORIF ORIF	24 27	8% 0%	NA
Turhan et al.	Retrospective	AORIF red ORIF	21 26	0% 0%	NA
Bi- and trimalleolar fractures					
Baumbach et al.	Prospective	AORIF ORIF	25 25	12% 8%	0.794
Ceccarini et al.	Retrospective	AORIF ORIF	38 75	0% 0%	> 0.05
Chiang et al.	Retrospective	AORIF red ORIF	65 40	8% 28%	**0.006**
Chou et al.	Retrospective	AORIF ORIF	22 27	14% 23%	0.488
Danilkowicz et al.	Retrospective	AORIF ORIF	11 11	9% 9%	NA
Smith et al.	Retrospective	AORIF ORIF	71 142	8% (AORIF + ORIF)	NA

Data marked as not available (NA). RCT = randomized controlled trial

**Table 5:** Operative time compared between AORIF and ORIF

	Operative time			
	AORIF	ORIF	Delay	p-value
Unimalleolar fractures				
Fuchs et al.	74 min	59 min	15 min	*p* = 0.027
Ge et al.	52.1 min ± 9.8	74.1 min ± 9.2	22 min	*p* = 0.000
Liu et al.	51 min ± 7	39 ± 5	12 min	*p* < 0.001
Turhan et al.	54 min ± 8.4	34 min ± 6	20 min	*p* < 0.0001
Bi-/Trimalleolar fractures				
Chiang et al.	105 min ± 27	94 min ± 23	11 min	*p* = 0.038
Chou et al.	145.9 min ± 32.3	157.7 min ± 53.9	− 11.8 min	*p* = 0.370
Danilkowicz et al.	105 min	74.18 min	31 min	*p* = 0.0036
Smith	89 min	79 min	10 min	*p* = 0.065

## Supplementary Information

Below is the link to the electronic supplementary material.

Supplementary Material 1

## Data Availability

No datasets were generated or analysed during the current study.
