# Supplementary material for: Arthroscopically assisted (AORIF) ankle fracture treatment seems to lead to superior results when compared to open reduction and internal fixation (ORIF) only: results of a systematic review
Source: Arch Orthop Trauma Surg. 2025 Sep 18;145(1):451. doi: 10.1007/s00402-025-06030-4 (PMC12446403; doi:10.1007/s00402-025-06030-4)
Supplement: Supplementary file 1 — Supplementary Material 1 [file 402_2025_6030_MOESM1_ESM.docx]

**Suppl. Table 1** Level of evidence by Wright et al. 2003 [1]. MINORS Tool, the items are scored 0 (not reported), 1 (reported but inadequate) or 2 (reported and adequate). The global ideal score being 16 for non-comparative studies and 24 for comparative studies [2].

|  |  | **1.** | **2.** | **3.** | **4.** | **5.** | **6.** | **7.** | **8.** | **9.** | **10.** | **11.** | **12.** |  |
| --- | --- | --- | --- | --- | --- | --- | --- | --- | --- | --- | --- | --- | --- | --- |
| **Author and Year** | **Level of evidence** | **A clearly stated aim** | **Inclusion of consecutive patients** | **Prospective collection of data** | **Endpoints appropriate to the aim of the study** | **Unbiased assessment of the study endpoint** | **Follow-up period appropriate to the aim of the study** | **Loss to follow up less than 5%** | **Prospective calculation of the study size** | **An adequate control group** | **Contemporary groups** | **Baseline equivalence of groups** | **Adequate statistical analyses** | **Score** |
| **Baumbach 2021** | **III** | 2 | 2 | 0 | 2 | 2 | 2 | 2 | 0 | 2 | 2 | 2 | 2 | **20** |
| **Ceccarini 2021** | **III** | 2 | 2 | 0 | 2 | 2 | 2 | 2 | 0 | 2 | 2 | 2 | 2 | **20** |
| **Chiang 2019** | **III** | 2 | 2 | 0 | 2 | 2 | 2 | 2 | 0 | 2 | 2 | 2 | 2 | **20** |
| **Chou 2023** | **III** | 2 | 2 | 0 | 2 | 2 | 2 | 2 | 0 | 2 | 2 | 2 | 2 | **20** |
| **Denilkowicz 2021** | **III** | 2 | 2 | 0 | 2 | 2 | 2 | 1 | 0 | 2 | 2 | 2 | 2 | **19** |
| **Fuchs 2016** | **III** | 2 | 2 | 0 | 2 | 2 | 2 | 2 | 0 | 2 | 2 | 2 | 2 | **20** |
| **Ge 2017** | **I** | 2 | 0 | 2 | 2 | 2 | 2 | 0 | 0 | 2 | 2 | 2 | 2 | **18** |
| **Liu 2020** | **II** | 2 | 2 | 2 | 2 | 2 | 2 | 2 | 2 | 2 | 2 | 2 | 2 | **24** |
| **Smith 2020** | **III** | 2 | 1 | 0 | 2 | 2 | 2 | 1 | 0 | 2 | 2 | 2 | 2 | **18** |
| **Takao 2004** | **I** | 2 | 0 | 2 | 2 | 2 | 2 | 0 | 0 | 2 | 2 | 2 | 2 | **18** |
| **Thordarson 2001** | **I** | 2 | 0 | 1 | 2 | 2 | 2 | 0 | 0 | 2 | 2 | 2 | 2 | **17** |
| **Turhan 2012** | **III** | 2 | 0 | 0 | 2 | 2 | 2 | 0 | 0 | 2 | 2 | 2 | 2 | **16** |

**Literature Suppl. Table 1**

1. Wright, J.G., M.F. Swiontkowski, and J.D. Heckman, *Introducing levels of evidence to the journal.* J Bone Joint Surg Am, 2003. **85**(1): p. 1-3.

2. Slim, K., et al., *Methodological index for non-randomized studies (minors): development and validation of a new instrument.* ANZ J Surg, 2003. **73**(9): p. 712-6.
